# Gas valves, forests and global change: a commentary on Jarvis (1976) ‘The interpretation of the variations in leaf water potential and stomatal conductance found in canopies in the field’

**DOI:** 10.1098/rstb.2014.0311

**Published:** 2015-04-19

**Authors:** David J. Beerling

**Affiliations:** Department of Animal and Plant Sciences, University of Sheffield, Sheffield S10 2TN, UK

**Keywords:** atmospheric carbon dioxide, earth system science, stomata, vegetation–climate feedbacks, water vapour

## Abstract

Microscopic turgor-operated gas valves on leaf surfaces—stomata—facilitate gas exchange between the plant and the atmosphere, and respond to multiple environmental and endogenous cues. Collectively, stomatal activities affect everything from the productivity of forests, grasslands and crops to biophysical feedbacks between land surface vegetation and climate. In 1976, plant physiologist Paul Jarvis reported an empirical model describing stomatal responses to key environmental and plant conditions that predicted the flux of water vapour from leaves into the surrounding atmosphere. Subsequent theoretical advances, building on this earlier approach, established the current paradigm for capturing the physiological behaviour of stomata that became incorporated into sophisticated models of land carbon cycling. However, these models struggle to accurately predict observed trends in the physiological responses of Northern Hemisphere forests to recent atmospheric CO_2_ increases, highlighting the need for improved representation of the role of stomata in regulating forest–climate interactions. Bridging this gap between observations and theory as atmospheric CO_2_ rises and climate change accelerates creates challenging opportunities for the next generation of physiologists to advance planetary ecology and climate science. This commentary was written to celebrate the 350th anniversary of the journal *Philosophical Transactions of the Royal Society*.

## 1. Introduction

**Figure 1.**
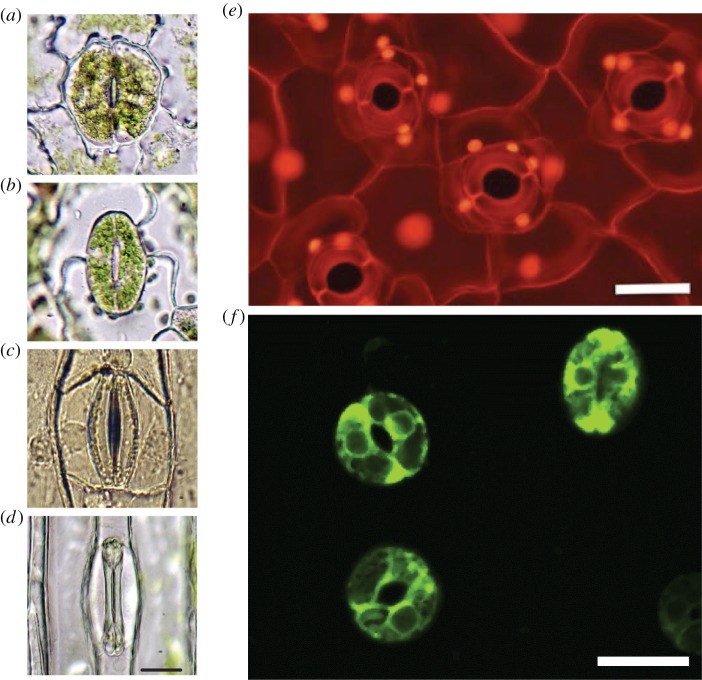
Diversity of stomata across the land plant kingdom. (*a*) The lycophyte *Huperzia prolifera*, (*b*) the fern *Nephrolepis exaltata*, (*c*) the herbaceous angiosperm *Tradescantia virginiana* and (*d*) the grass (wheat) *Triticum aestivum*. (*e*) Images of stomata on a leaf epidermis of *Commelina communis* showing fully inflated guard cells creating approximately circular open pores (scale bar, 50 µm). (*f*) Images of fluorescing stomata with guard cells expressing guard cell specific GFP-tags (green fluorescent proteins) on a leaf epidermis of *Arabidopsis* (scale bar, 25 µm) (S. Casson, University of Sheffield, unpublished). Images (*a*–*d*) reprinted with permission from Franks & Farquhar [1] (Copyright © American Society of Plant Biologists), (*e*) reproduced with permission from Franks *et al.* [2].

Stomata are microscopic pores on the surfaces of leaves (called stomata after the Greek for mouth, *stoma*). Each individual stoma is typically composed of two specialized guard cells that flank an adjustable aperture and regulate the inevitable escape of water vapour as leaves take up CO_2_ for photosynthesis (figure 1): inevitable because to assimilate CO_2_ from the atmosphere and synthesize biomass by photosynthesis, plants must open their stomatal apertures, exposing the wet surfaces of the photosynthetic cells inside the leaf to the drier atmosphere. Consequently, the business of building plants from atmospheric CO_2_ is expensive in terms of water requirements. For example, on average it typically requires approximately 1 kg of water to synthesize every 2–6 g of plant dry matter depending on weather conditions, especially atmospheric dryness, and the photosynthetic mode of the plants [3].

**Figure 2.**
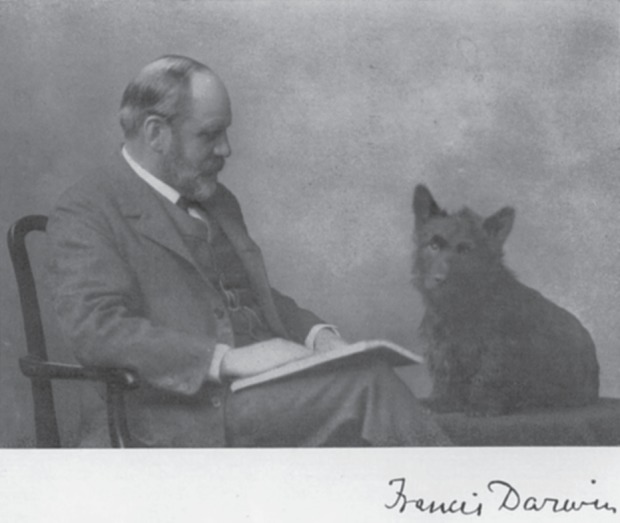
Francis Darwin FRS with his canine companion Scrubbins (from [4]). Copyright © The Royal Society.

Plant physiologists have a long history of reporting discoveries concerning the behaviour of these fascinating structures in the *Philosophical Transactions of the Royal Society*, reaching back over a century to the pioneering work of Sir Francis Darwin FRS (1848–1925), the third son of Charles Darwin FRS (1809–1882) (figure 2). Darwin [5] was broadly interested in the control of water loss from leaves experiencing variations in irradiance and atmospheric dryness. His major contribution to stomatal research arose from inventing, and then exploiting, equipment to make ground-breaking quantitative measurements of the effects of environmental factors, and plant water status, on the apertures of these tiny gas valves [6,7]. Before Darwin, it was established that atmospheric CO_2_ entered leaves through stomatal pores and water escaped through them in the transpiration stream by evaporation [5,8]. After Darwin's work, it became clear that stomatal apertures responded sensitively to changing environmental conditions and regulated the transpiration rates of leaves [5,8].

**Figure 3.**
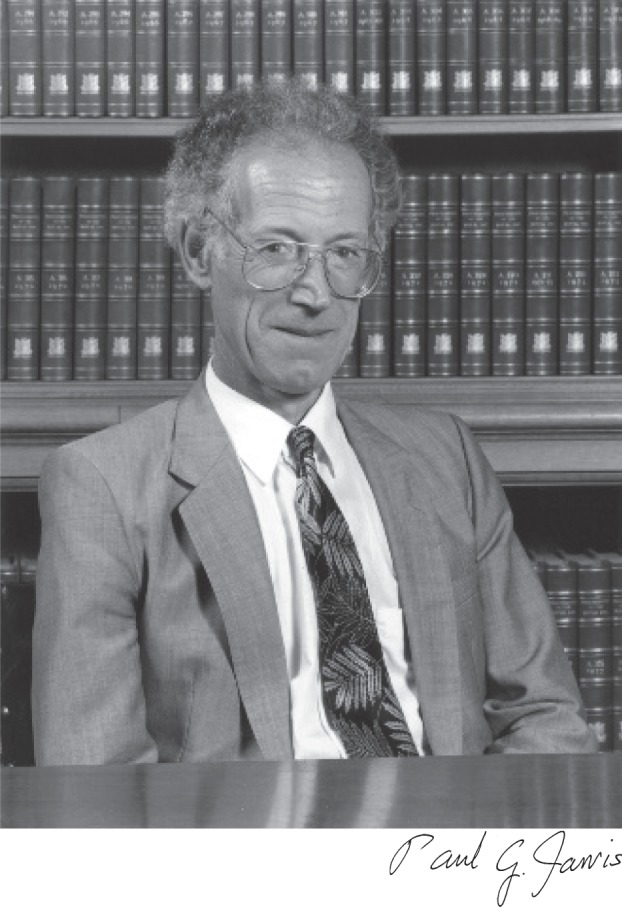
Paul Jarvis FRS in 1997. Copyright © The Royal Society.

These research themes echo those of Paul Jarvis FRS (1935–2013) (figure 3). Jarvis and McNaughton [9,10] investigated the environmental responses of stomata and went on pioneer the scientific analysis of how their collective actions on individual leaves translated to affect the exchange of water vapour, CO_2_ and energy between forest canopies and the atmosphere. Born in Tunbridge Wells, Kent (in common with the author), Jarvis was the son of a Hertfordshire farmer who was also a founder member of the Royal Air Force Regiment in World War II [11]. His mother was a secretary to the statistician and geneticist Karl Pearson FRS (1857–1936) at University College, London [11]. Jarvis's scientific career began when he read Botany at Oriel College, Oxford, before undertaking post-graduate research investigating the limits to the distribution of oaks and other tree species in the UK at the University of Sheffield, where coincidentally, Francis Darwin was made an Honorary Doctor of Science in 1910 [4]. Following productive spells in Sweden and Australia, Jarvis became Professor of Forestry and Natural Resources at the University of Edinburgh in 1975. Jarvis's subsequent long and distinguished research career ‘laid the foundations for decades of studies on the interplay between forests and the climate system’ [11].

Fittingly, it was Jarvis's studies modelling the behaviour of stomata under changing environmental conditions and plant water status that formed the subject of his *Philosophical Transactions of the Royal Society* paper nearly 40 years ago [12]. It was read during the two-day 1975 Royal Society Discussion Meeting ‘A Discussion on Water Relations of Plants', which was described as the ‘first major meeting on plant water relations to be held in Britain since 1964’ [13]. Ironically, the meeting took place at a time when the UK was suffering the most severe heatwave in more than 350 years, with widespread drought, tree mortality and devastating crop failure. A modern metric of the article's significance to the field is given by the cumulative number of times it has been cited in the scientific literature, as recorded in Thompson Reuters Web of Science. Currently (as of May 2014), this figure stands at over 1400, and the article continues to attract 70–90 citations per year, nearly 40 years after its publication. Here a brief introduction to Jarvis [12] is provided, together with some historical background, and a commentary on its significance. The scope then widens to say something about how the subject evolved over subsequent decades to inform debates concerning the uncertain future of the Earth's biota and climate in the coming century.

## 2. Capturing stomatal behaviour with equations

The pioneering contribution made by Jarvis [12] was deceptively simple. It introduced plant physiologists to a simple mathematical approach for describing how stomata respond to changes in the environment and plant water status to affect rates of water loss from leaves. The model had its genesis in a project Jarvis led studying coniferous forests in Aberdeenshire, North East Scotland, and was really the first attempt to apply the methods and emerging techniques of ‘environmental physiology’ to a forest community, in this case a Sitka spruce plantation. In developing it, Jarvis took a series of disparate measurements on leaves and made sense of them with a unifying explanatory empirical model relevant to plant biologists, crop scientists, foresters and meteorologists.

Capturing stomatal behaviour mathematically to model the flux of water vapour from leaves necessitates first describing that behaviour with measurements—itself no easy task. Water loss through stomata is commonly expressed as leaf stomatal conductance (denoted *g*_s_, or its inverse, stomatal resistance, *r*_s_). In other words, the conductance of stomata to the passage of water vapour from the water-saturated leaf interior to the drier free-air immediately surrounding the outside of the leaf. Up until around the mid-twentieth century, plant physiologists commonly studied stomatal behaviour with a mass flow porometer, an instrument based on the porometer invented by Darwin & Pertz [14]. Mass flow porometers measure the flow of air into and out of a leaf due to an applied pressure gradient. The main pathway of air flow is through the stomata on one side of the leaf across the intercellular airspaces of the tissues and out of stomata on the other side. This means the volume flow rate depends on the series resistances of two epidermes and the intercellular air spaces of the leaf mesophyll. So reliable measurements can really only be made on leaves with similar numbers of stomata on the upper and lower surfaces. Complexities of measurements with mass flow porometers are numerous [15]. A major problem is the difficulty of relating the measurements to *g*_s_ because the technique measures the viscous flow resistance through stomata, whereas water vapour exchanges are largely diffusive [16].

**Figure 4.**
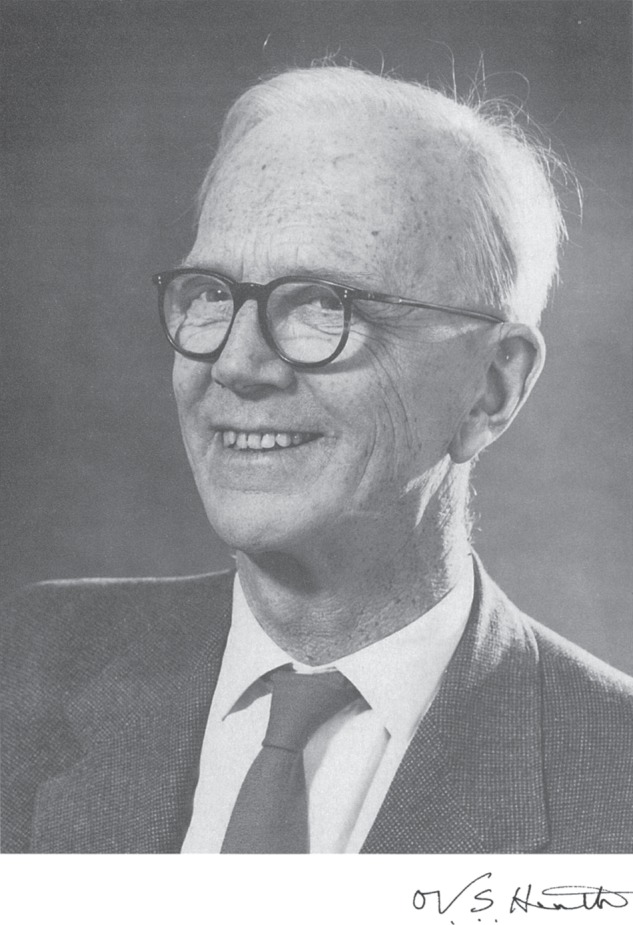
Oscar Victor Sayer Heath FRS.

Development of a more sophisticated device called a diffusion porometer followed with the advent of electronic water vapour sensors to accurately sense humidity, and improved construction materials. These instruments allowed measurement of the stomatal conductance (or resistance) of leaves to water vapour transfer (e.g. [17,18]). Concurrent measurements of the rate of CO_2_ assimilation of leaves were also emerging from laboratories using infrared gas analysers (IRGAs) [19], but these were less than routine and involved complex instrumentation that required careful maintenance. Nevertheless, careful laboratory-based investigations exploiting advances in diffusion porometers, including decisive and meticulous experiments by O.V.S. Heath FRS (1903–1997) (figure 4) [20,21], and other distinguished scientists (see [8] for a review), established that the stomatal conductance of leaves responded sensitively to changes in four important environmental variables: irradiance, atmospheric CO_2_ concentration, temperature and atmospheric dryness. Stomata were additionally known to respond to changes in the water status of soils and plant tissues, as defined by the soil water potential and leaf water potential, respectively, a point emphasized by Jarvis [12]. To complicate things further, each of these variables interacted with the other to determine the resulting steady-state stomatal conductance of leaves.

Jarvis's [12] solution to the tricky problem of modelling the stomatal conductance of leaves for a given set of environment conditions, developed through his acknowledged collaboration with Dr K.L. Reed at the Department of Natural Resources, Forest Land Management Centre, Washington [22], was as follows: ‘The simplest hypothesis, which we have adopted, is that the stomatal conductance of leaves is the result of complete expression of the influence of all the variables without any synergistic interactions’ (p. 603). The outcome of the assumption was built into a simple equation where the resulting *g*_s_ value of a leaf was the product of the five environmental variables listed above normalized to the minimum stomatal conductance of the leaf. It was illustrated with the following example. ‘That is to say, if *g*_s_ is reduced to 80% of its maximum by the prevailing photon flux (irradiance) and to 80% of the maximum by the prevailing temperature, the results *g*_s_ will be 64% of the maximum *g*_s_ value’ [12, p. 605]. Before cautiously remarking, ‘Further experiments are needed to show whether this hypothesis is adequate’.

Having established the basis of the approach, the rest of the paper analyses new measurements, or datasets from field or laboratory grown plants, including from the Aberdeen Sitka spruce project [23,24]. Obtaining these field measurements of *g*_s_ was a difficult undertaking, and they were often made with equipment built by Jarvis's team. Joe Landsberg, one of the scientists involved, recalls ‘it was rare to get a few days’ good data without breakdowns'. The laboratory system in Aberdeen for measuring the gas exchange of foliage ‘was a wondrous collection of pumps, mixing valves and flow meters, drying columns, CO_2_ bottles, water baths for temperature control, lights that generated considerable heat, so fans to cool the system, and of course the gas analyser and humidity measuring equipment. All this was focused on producing precisely controlled conditions in a small Perspex chamber containing spruce shoots, and measuring the properties of the air flowing into and out of that chamber’ (Landsberg J, 2014 personal communication).

Datasets collected with these sorts of equipment generated relationships between *g*_s_, leaf water potential and the four environmental variables (temperature, light, atmospheric moisture and atmospheric CO_2_ concentration) that were then described by equations ‘fitted’ to the data. Part of this process involved ‘fitting’ lines to the upper limit of the observations. These boundary lines proved useful to delimit the maximum values of *g*_s_ for a given set of environmental conditions. Unfortunately, no archive material remains documenting quite what the referees and editor thought of this idea. But Jarvis's method of drawing an upper boundary line over a scatter of data points was the source of some amusement to his colleagues, as John Grace his colleague at the University of Edinburgh recalls. ‘Reviewers must have pointed out that the less than rigorous nature by which these lines were drawn but it turned out to be a useful way forward. Henceforth we called them Jarvisian Envelopes’ (Grace J, 2014 personal communication). Jarvis [12] finishes by testing the approach with extensive datasets of *g*_s_ on shoots of Sitka spruce (*Picea sitchensis*) trees in the UK and shoots of Douglas fir (*Pseudotsuga menziesi*) trees in the USA; all were measured with the diffusion porometer technique. For trees at both sites, environmental datasets were available, with measurements of irradiance, temperature, vapour pressure deficit and so on, for driving the model. The model successfully explained 51 and 73% of the observed variation in *g*_s_ values at the Sitka spruce and Douglas fir sites, respectively, with values of parameters derived from the model being rather different between the sites. For example, Sitka spruce needles had higher maximum *g*_s_ values and responded more sharply to increasing irradiance than the Douglas fir needles. Jarvis commented that ‘These differences in parameters may result from differences between the species, but more probably describe the differences in the physiological condition of the trees in spring and autumn at the two sites’ [12, p. 607].

At the 1976 Royal Society Meeting in London, the paper seemed to go over the heads of most of the audience with the exception of ‘old Penman [Howard Penman FRS (1909–1984), British meteorologist], who jumped up and said Paul's model was nonsense’ (Linder S, 2014 personal communication) recalls Sune Linder of the Southern Swedish Forest Research Centre. The comment perhaps reflects the dichotomy between a physicist's and botanist's view of how the world works and expectations for how it should be described. Jarvis was, however, the first to recognize the short-comings of his empirical approach and its preliminary nature. He wrote ‘Interpreting the response of stomata to environmental variables in this way is practically useful, in that the parameters can be used to make predictions, but it is not wholly satisfactory. The parameters have limited physiological meaning because the model is descriptive rather than mechanistic’ [12, p. 609]. In the years that followed, the Jarvis model was widely applied mainly at the leaf and canopy level. Whitehead *et al.* [25], for example, measured stomatal responses to environmental variables in the field of tropical tree species in Nigeria and followed Jarvis's approach in fitting the conductance values to environmental variables. Others have used and successful tested the approach, with modification, and applied it to *Eucalyptus* [26], *Populus* [27] and *Picea* [28].

## 3. Towards planetary ecology

Two major scientific advances followed. First was the development of a mathematical theory showing that leaves trade carbon for water in such a way as to maximize carbon gain with respect to water loss over time [29], stomata, of course, being the central decision takers making the soil water-for-atmospheric carbon trading scheme of this emerging paradigm a success. Second, technological developments led to advances and miniaturization of IRGAs for making gas exchange measurements on leaves. IRGAs became small enough to build into portable field systems that allowed control and measurement of water vapour and CO_2_ concentrations in air streams entering and exiting leaf cuvettes, to routinely make simultaneous measurements of CO_2_ assimilation rates and stomatal conductance. Empirical studies of plants exploiting emerging IRGA gas exchange system technology and theory revealed that stomatal conductance covaried with photosynthesis, with one feeding back to affect the other [30]. Accurately modelling stomatal conductance, it turned out, now meant modelling leaf photosynthesis too [31].

**Figure 5.**
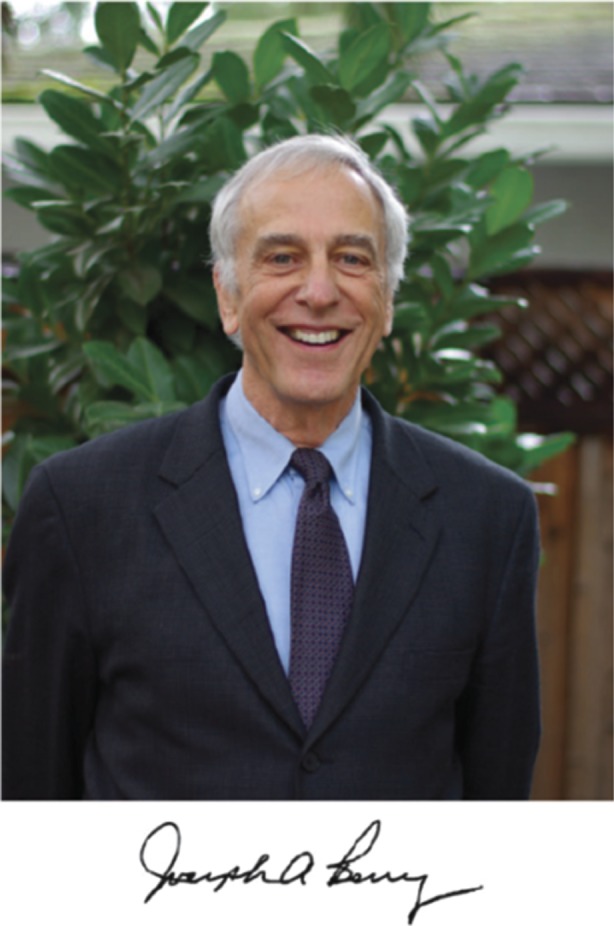
Joseph Berry of the Carnegie Institution for Science at Stanford in 2011 (image provided by Joseph Berry).

That development followed when Graham Farquhar FRS together with Susanne von Caemmerer, both at the Australian National University (ANU), and Joe Berry at the Carnegie Institute, Stanford (figure 5) developed a mathematical model accurately simulating the photosynthesis of leaves under a wide range of natural conditions [32]. Their biochemical model of leaf photosynthesis is itself now heavily cited (above 3300, Web of Science, May 2014) and continues to accumulate over 200 citations annually. It provided an eloquent and mechanistically sound approach for simulating the photosynthetic carbon uptake of leaves in response to a range of environmental conditions. An update on the continued development and application of this photosynthesis model, including larger scale modelling and remote-sensing applications, is given in Bernacchi *et al.* [33].

But the development of a robust photosynthesis model exposed a problem. Existing stomatal conductance models like that described by Jarvis [12], and others, did not work well when combined with a photosynthesis model and this called for a rethink in how the problem was approached. Joe Berry, a world-leading stomatal physiologist, together with his then graduate student Tim Ball, and their colleague Ian Woodrow, did this by analysing hundreds of IRGA-derived gas exchange measurements. Here Berry [34] describes in his own words what happened next.

Drawing on an abundance of careful gas exchange measurements from Chin Wong's PhD thesis [at ANU] and additional measurements of his own, Tim Ball found that a regression including the rate of photosynthesis as one of the variables controlling conductance provided an excellent fit to hundreds of independent observations of conductance spanning several species and a wide range of environmental conditions. (p. 8)

The empirical equation that Ball *et al.* [35] formulated from these analyses did a remarkably good job of predicting the stomatal conductance of leaves and was underpinned by detailed analyses of the degree to which the leaf boundary layer, stomata and the primary carboxylation enzyme, Rubisco, determine the rate of photosynthesis [36,37]. In the ‘Ball–Woodrow–Berry (BWB) model’, as the comment above suggests, *g*_s_ is linked to photosynthesis. This linkage provides an elegant means of accounting for the complex environmental and biological control of stomatal conductance by light, temperature and plant species, each plant species having its own particular physiological characteristics. As before, the atmospheric CO_2_ concentration and humidity needed to be accounted for in a species-specific manner and were dealt with using species-specific regression term as additional controlling variables [34]. Berry adds ‘Of course, one would still need to predict the rate of photosynthesis (itself a function of stomatal conductance) to predict conductance using this ‘Ball–Woodrow–Berry equation,’ but this is accomplished using the biochemical model and a straightforward numerical approach …. This combination of models made it possible to accurately simulate photosynthesis and transpiration of leaves in natural environments’ [34, p. 8]. The ‘straightforward numerical approach’ alluded to here is really finding iterative solutions to sets of nonlinear coupled leaf photosynthesis and stomatal conductance equations; analytical solutions were later developed [38]. It should not be lost, however, that the new BWB solution to the problem drew heavily on the original approach developed by Jarvis [12]. But it had the advantage of scaling stomata conductance as a function of photosynthesis, regardless of which environmental resources (e.g. soil water and nitrogen) constrained that process.

Having captured the responses of stomata with equations, the next step was to think about modelling how they influenced the feedback of the Earth's land surface vegetation on regional and global climate in a high-CO_2_ ‘greenhouse’ world. The rationale for this idea originated half a century ago with fundamental stomatal research by O.V.S. Heath [39] (figure 4). Rapidly rising global atmospheric CO_2_ concentrations since pre-industrial times brought about by burning fossil fuels not only affect climate, via the greenhouse effect, but also, as Heath demonstrated, cause the stomata of many plant species to close partially. The distinguished stomatal physiologist T.A. Mansfield FRS explains the significance of these observations for climate change in Heath's obituary written for *The Independent* newspaper (24 June 1997) ‘This alters the rate of transfer of water from the soil to the atmosphere, and it also affects the surface-atmosphere exchange of heat and contributes to global warming. Thus the ability of stomata to sense and respond to CO_2_ in the atmosphere, once thought to be an obscure topic only of academic interest to Heath and a few other scientists, has become a major factor in our understanding of the forces that are driving climate change’.

Berry [34] recalls the major problem with investigating this CO_2_-linked stomata–vegetation–climate feedback before the BWB stomatal conductance model was incorporated into interactive terrestrial vegetation models:

[in the model] … plants on the land opted to conserve water, which caused the atmosphere to dry and resulted in less rain and warmer temperatures, which caused the plants to try even harder to conserve water. The result was a condition Dave Randall [a collaborating climate modeller] described as ‘stomatal suicide,’ where the land masses of the planet became deserts. (pp. 9–10)

Implications of an improved numerical representation of stomatal behaviour for modelling biosphere–atmosphere interactions in a high-CO_2_ world soon became apparent. In the mid-1990s, a team of North American scientists, including Berry, successfully coupled the BWB model into a land surface scheme nested within a model of the global climate system [40]. The team included the UK-born NASA meteorologist Piers Sellers, who went on to forge a career as an astronaut and flew three missions on the space shuttles *Atlantis* (2002, 2010) and *Discovery* (2006), and is now back in science working as the Deputy Director of Science and Exploration at NASA's Goddard Space Flight Centre, Maryland. Sellers knew Jarvis well. He'd been taught through lectures by him during the final year of his degree course in ecological sciences at the University of Edinburgh, and later worked closely with him on a large international field experiment on the Canadian boreal forests [41].

**Figure 6.**
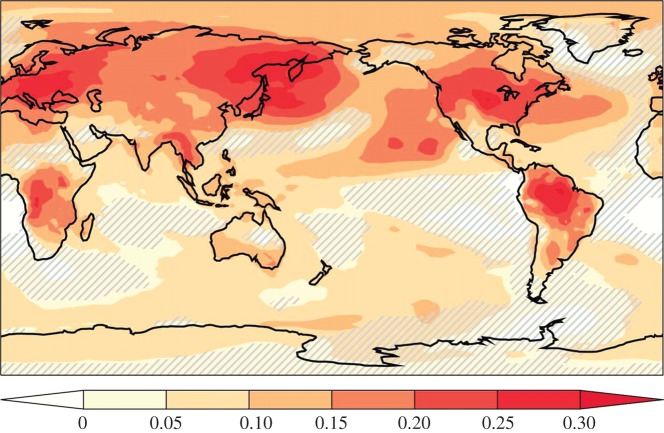
Fraction of total surface warming (i.e. warming caused by the combined radiative and physiological effects) associated with the physiological effects of CO_2_ on stomatal behaviour. Results displayed were obtained in a climate model simulation with double present-day atmospheric CO_2_ concentration (800 ppm). Hatched areas indicate regions not statistically significant at the 5% level using the Student *t*-test. (Image from [42].)

In their *Science* paper, Sellers *et al.* [40] reported findings from numerical model experiments designed to address two fundamental questions: (1) what effect does doubling the atmospheric CO_2_ concentration have on stomatal conductance and (2) what is its feedback on regional and global climate? The results showed that CO_2_-driven reductions in canopy transpiration exerted marked warming in tropical regions by +0.9°C owing to reductions in latent heat transfer. Put another way, this physiological forcing of climate, as it became known, contributed about half of the overall 1.7°C warming caused by the radiative effects of a doubled CO_2_ atmosphere itself in these areas (i.e. trapping of long-wave radiation). Subsequent detailed land carbon cycle–climate system modelling simulation studies reported that partial stomatal closure in response to a doubling in the atmospheric CO_2_ concentration causes additional warming across approximately 20% of the land surface, including large areas of the boreal forest and the tropical forests in South America and Africa [42]. In these heavily vegetated regions, the physiological forcing of climate accounted for up to 30% of the total warming, i.e. that caused by plant physiology and an enhanced greenhouse effect (figure 6). Less widely open stomata reduced canopy transpiration rates and the latent heat flux, and also decreased low cloud cover. Less clouds reduced the planetary albedo and meant more solar radiation could warm the Earth's surface [42]. The magnitude of such vegetation–climate feedbacks is uncertain and difficult to establish and will probably depend on the time scale involved. Changes in vegetation structure (e.g. leaf area index, LAI) and distribution could exert complicating effects. If elevated CO_2_ increases canopy LAI, it could offset the partial stomatal closure of leaves and increase canopy transpiration in some regions of the world [43]. Nevertheless, it is now well-established that vegetation responses to CO_2_ and climate change could feedback to influence climate; development of the scientific thinking behind modelling land surface–climate interactions with a strong biophysical perspective is given by Sellers *et al.* [44].

Land carbon cycle models currently simulate a wide range of processes to predict changes in the productivity and net exchange of CO_2_, water vapour and energy between terrestrial ecosystems and the atmosphere (e.g. [45–47]). In consequence, these global models are key tools for investigating the behaviour of the land carbon sink under given future CO_2_ and climate change scenarios [48,49]. The land carbon ‘sink’ referred to here is that created by forests as they remove CO_2_ out of the atmosphere to synthesize leaves, wood and roots, some of which can also be released back to the atmosphere by agents of disturbance, especially fire. Although the sink varies from year to year, on average it soaks up one-quarter of the annual CO_2_ emissions from the burning of fossil fuels [48,49], raising the question: what will happen in the future? Many of the current generation of land carbon cycle models addressing this point still largely simulate stomatal conductance responses to the environment by adopting the empirical BWB or similar approach [49]. Mechanistic models that aim to describe how stomata function are being developed, but are limited by our poor understanding of the underlying complexity [50]. Optimization models, another class of models in development, revisit the ideas [29], in the hope that they might provide useful insights into why plants behave as they do when environmental conditions change and improve future carbon cycle predictions [51,52].

Nevertheless, it is a telling situation that the current generation of terrestrial carbon cycle models used in the 5th Assessment Report of the Intergovernmental Panel on Climate Change often underestimate the productivity of vegetation in water-limited regions, a feature highlighting the need for better representation of plant–soil processes in global models [49]. Many aspects of plant water use are linked to changes in the hydraulic pathway from roots to the canopy, with plant water potential regulated by stomata to maximize water uptake and avoid breaking hydraulic contact with the soil water [53]. Here too Jarvis [12] was prescient. He proposed a mechanistic model linking the leaf water potential to the resistance pathway water encounters as it moves from the soil to the canopy. From this, a second equation followed, making leaf water potential dependent on evaporation rate that incorporated the interactive effects of light, temperature and atmospheric dryness. In this way, he showed evaporation rates from Sitka spruce and Scots pine forests show close linear relations with leaf water potentials.

Stomata themselves respond to water stress through increases in the abscisic acid (ABA) concentration of the sap carried through the xylem from the roots, and this induces stomatal closure and slows transpiration [54]. This discovery saw the BWB approach modified empirically to account for xylem ABA effects on stomatal conductance in a manner mirroring Jarvis's approach [55]. The end-result is more realistic modelling of the plant–soil hydraulic pathway, so that as plant transpiration dries the soil, leaf stomatal conductance drops. These and other developments are reviewed by Buckley & Mott [50]. Exploration of alternative formulations capturing the optimality behaviour of stomata [29] has also proved promising for simulating tropical forest water fluxes [56], as have implementations of more detailed plant hydraulic systems in a global vegetation model [57]. Nevertheless, improving plant–soil water linkages, among other neglected processes like phosphorus cycling and microbial decomposition, is a frontier in ecosystem modelling [49,58].

Process-based modelling of terrestrial ecosystems has progressed in parallel with the development of crop models, but crop modellers have been slow to incorporate models of stomatal conductance or couple them with a model of photosynthesis to predict yields and food supply worldwide [59]. Instead, models tend to rely on outdated and potentially misleading CO_2_ ‘fertilization factors’ that may overestimate crop yields under elevated atmospheric CO_2_ concentrations [60]. These same models also tend to omit the effects of atmospheric CO_2_ on stomatal closure, soil moisture and canopy temperature that free-air CO_2_ enrichment (FACE) studies have shown to be important [60,61]. FACE systems allow for the natural coupling of crops and the atmosphere, and crops grown within FACE systems typically show 5–15% decreases in canopy transpiration and crop water use [62]. Obviously, mechanistically modelling crop yields in response to future CO_2_ and climate change scenarios, including the interactive effects of surface ozone, temperature, moisture and light, is an important goal. Addressing this challenge requires improved representation of stomatal physiology to better link crops with the soil and atmospheric environments [59].

## 4. Retrospective

Looking back, it is clear that Jarvis [12] presciently anticipated the need to model stomatal behaviour in response to a range of environmental factors. His paper proposed roles for stomata in regulating the gas exchange of forest shoots and canopies that proved important for determining critical aspects of vegetation–atmosphere interactions. He proposed a framework for attempting this at a time when modellers of the Earth's global climate system had not yet begun to recognize (let alone incorporate) the feedback of vegetation on the global cycles of carbon, water and energy. Within a decade, other research groups, notably those in North America and Australia, advanced the theory and developed refined models that established the current paradigms for understanding the behaviour of stomata. Most recently, molecular genetic controls on stomatal development [63], CO_2_ sensing [64] and regulation of formation by environmental variables like atmospheric CO_2_ [65] have been elucidated, allowing integration with leaf gas exchange properties [66–68].

Assessment of feedbacks involving stomata, forests and climate in future high-CO_2_ ‘greenhouse’ worlds continues [49], with the wider implications of Heath's seminal observations concerning partial stomatal closure under high CO_2_ proving a challenge to understand. Recent atmospheric CO_2_ increases have reduced transpiration rates from temperate and boreal forest canopies in this way to a far greater degree than sophisticated ecosystem models anticipated [69]. Reduced canopy transpiration means less water is taken up by roots, with more remaining in the soil to affect the water balance of the land surface. Through this mechanism, the ‘anti-transpirant’ effect of a rising atmospheric CO_2_ concentration is now invoked to explain (over and above other factors) increased land surface run-off from major river basins since the 1960s [70].

In his closing remarks to the 1976 Royal Society Meeting, Monteith [13] quoted the Victorian poet Alfred, Lord, Tennyson's lines written at the time of the potato famine in Ireland:

Science moves but slowly, slowly, creeping on from point to point Slowly comes a hungry people, as a lion creeping nigher,

Glares at one that nods and winks behind a slowly dying fire. [13, p. 612]

Monteith [13] creatively interpreted the modern relevance of these lines to ask ‘whether we are the people nodding and winking behind sophisticated research projects while hunger and malnutrition remain an immense global problem’. Decades later the growing realization is that agricultural food production needs to double by 2050 to keep pace with the expanding population of humans [71,72] and this urgent challenge is set against a background of rising atmospheric CO_2_ concentrations and changing climate [73]. Improving our ability to feed a global population of 9 billion hungry humans, and model planetary ecology and climate [74], demands an improved understanding of complex stomatal physiology. What follows next will build on the scientific foundations laid by Paul Jarvis nearly four decades ago [12].

## Author profile

**David Beerling** is the Sorby Professor of Natural Sciences at the University of Sheffield. His leads an interdisciplinary research group investigating fundamental questions concerning the coevolution of terrestrial ecosystems and the global environment over the past half billion years. He has published numerous articles in learned academic journals and edited issues of both the Biological Sciences and Physical Sciences series of the *Philosophical Transactions of the Royal Society*. He authored the best-selling the popular science book *The Emerald Planet* (Oxford University Press, 2007), which argued the case for the role of plants in shaping the Earth's history. The book formed the basis of a major three-part BBC Two television series, *How to Grow a Planet*. He was elected to the Fellowship of the Royal Society in 2014.

## References

[1] Franks PJ Farquhar GD 2007 The mechanical diversity of stomata and its significance in gas-exchange control. Plant Physiol. 143, 78–87. (10.1104/pp.106.089367) 17114276 PMC1761988

[2] Franks PJ Leitch IJ Ruszala EM Hetherington AM Beerling DJ 2012 Physiological framework for adaptation of stomata to CO_2_ from glacial to future concentrations. Phil. Trans. R. Soc. B 367, 537–546. (10.1098/rstb.2011.0270) 22232765 PMC3248712

[3] Larcher W 2003 Physiological plant ecology. Ecophysiology and stress physiology of functional groups, 4th edn Berlin, Germany: Springer.

[4] Blackman FF 1932 Obituary notices. Francis Darwin—1848–1925. Proc. R. Soc. Lond. B 110, i–v. (10.1098/rspb.1932.0031)

[5] Ayres P 2008 The aliveness of plants: the Darwins at the dawn of plant science. London, UK: Pickering & Chatto.

[6] Darwin F 1898 Observations on stomata. Phil. Trans. R. Soc. Lond. B 190, 531–621. (10.1098/rstb.1898.0009)

[7] Darwin F 1916 On the relation between transpiration and stomatal aperture. Phil. Trans. R. Soc. Lond. B 207, 413–437. (10.1098/rstb.1916.0009)

[8] Meidner H 1987 Three hundred years of research into stomata. In Stomatal function (eds Zeiger E Farquhar GD Cowan IR), pp. 7–27. Stanford, CA: Stanford University Press.

[9] Jarvis PG McNaughton KG 1986 Stomatal control of transpiration—scaling up from leaf to region. Adv. Ecol. Res. 15, 1–49. (10.1016/S0065-2504(08)60119-1)

[10] McNaughton KG Jarvis PG 1991 Effects of spatial scale on stomatal control of transpiration. Agric. For. Meteorol. 54, 279–302. (10.1016/0168-1923(91)90010-N)

[11] Mencuccini M 2013 Paul Jarvis, FRS, FRSE: plant ecologist who showed the link between forests and the atmosphere. iForest 6, 100–101. (10.3832/ifor0102-006)

[12] Jarvis PG 1976 The interpretation of the variations in leaf water potential and stomatal conductance found in canopies in the field. Phil. Trans. R. Soc. Lond. B 273, 593–610. (10.1098/rstb.1976.0035) PMC4360119 25750234

[13] Monteith JL 1976 Closing remarks. Phil. Trans. R. Soc. Lond. B 273, 611–613. (10.1098/rstb.1976.0036)

[14] Darwin F Pertz DFM 1911 On a method of estimating the aperture of stomata. Proc. R. Soc. Lond. B 84, 136–154. (10.1098/rspb.1911.0058)

[15] Penman HL 1942 Theory of porometers used in the study of stomatal movements of leaves. Proc. R. Lond. Soc. B 130, 416–433. (10.1098/rspb.1942.0010)

[16] Parkinson KJ 1985 Porometry. Soc. Exp. Biol. Semin. Ser. 22, 171–191.

[17] Heath OVS Mansfield TA 1962 A recording porometer with detachable cups operating on four separate leaves. Proc. R. Soc. Lond. B 156, 1–13. (10.1098/rspb.1962.0024)

[18] Beardsell MF Jarvis PG Davidson B 1972 A null-balance diffusion porometer suitable for use with leaves of many shapes. J. Appl. Ecol. 9, 677–690. (10.2307/2401897)

[19] Moss DN Rawlins SL 1963 Concentration of carbon dioxide inside leaves. Nature 197, 1320–1321. (10.1038/1971320a0)

[20] Heath OVS 1948 Control of stomatal movement by a reduction in the normal [CO_2_] of the air. Nature 161, 179–181. (10.1038/161179a0) 18901741

[21] Heath OVS Russell J 1954 An investigation of the light responses of wheat stomata with the attempted elimination of control by the mesophyll. J. Exp. Bot. 5, 1–15. (10.1093/jxb/5.1.1)

[22] Reed KL Hamerly ER Dinger BE Jarvis PG 1976 An analytical model for field measurement of photosynthesis. J. App. Ecol. 13, 925–942. (10.2307/2402267)

[23] Landsberg JJ Beadle CL Biscoe PV 1975 Diurnal energy, water and CO_2_ exchanges in an apple (*Malus pumila*) orchard. J. App. Ecol. 12, 659–684. (10.2307/2402181)

[24] Watts WR Neilson RE Jarvis PG 1976 Photosynthesis in Sitka spruce (*Picea sitchensis*) (Bong.) Carr.) VII. Measurements of stomatal conductance and ^14^CO_2_ uptake in a forest canopy. J. App. Ecol. 13, 623–638. (10.2307/2401808)

[25] Whitehead D Okali DUU Fasehun FE 1981 Stomatal response to environmental variables in two tropical forest species during dry season in Nigeria. J. App. Ecol. 18, 571–587. (10.2307/2402418)

[26] White DA Beadle CL Sands PJ 1999 Quantifying the effect of cumulative water stress on stomatal conductance of *Eucalyptus globulus* and *Eucalyptus nitens*: a phenomenological approach. Aust. J. Plant Physiol. 26, 17–27. (10.1071/PP98023)

[27] Kim H-S Oren R Hinckley TM 2008 Actual and potential transpiration and carbon assimilation in an irrigated poplar plantation. Tree Physiol. 28, 559–577. (10.1093/treephys/28.4.559) 18244943

[28] Ward EJ Oren R Sigurdsson BD 2008 Fertilization effects on mean stomatal conductance are mediated through changes in the hydraulic attributes of mature Norway spruce trees. Tree Physiol. 28, 579–596. (10.1093/treephys/28.4.579) 18244944

[29] Cowan IR Farquhar GD 1977 Stomatal function in relation to leaf metabolism and environment. In Integration of activity in the higher plant (ed Jennings D), pp. 471–505. *Soc. Exp. Biol. Symp.* 31 Cambridge, UK: Cambridge University Press. 756635

[30] Wong SC Cowan IR Farquhar GD 1979 Stomatal conductance correlates with photosynthetic capacity. Nature 282, 424–426. (10.1038/282424a0)

[31] Farquhar GD Sharkey TD 1982 Stomatal conductance and photosynthesis. Annu. Rev. Plant Physiol. 33, 317–345. (10.1146/annurev.pp.33.060182.001533)

[32] Farquhar GD von Caemmerer S Berry JA 1980 A biochemical model of photosynthetic CO_2_ assimilation in leaves of C_3_ species. Planta 149, 78–90. (10.1007/BF00386231) 24306196

[33] Bernacchi CJ Bagley JE Serbin SP Ruiz-Vera UM Rosenthal DM VanLoocke A 2013 Modelling C_3_ photosynthesis from the chloroplasts to the ecosystem. Plant Cell Environ. 36, 1641–1657. (10.1111/pce.12118) 23590343

[34] Berry JA 2012 There ought to be an equation for that. Annu. Rev. Plant Biol. 63, 1–17. (10.1146/annurev-arplant-042811-105547) 22242962

[35] Ball JT Woodrow IE Berry JA 1987 A model predicting stomatal conductance and its contribution to the control of photosynthesis under different environmental conditions. In Progress in photosynthesis research (ed. Biggins J), vol. 4, pp. 221–224. Dordrecht, The Netherlands: Kluwer Academic Publishers.

[36] Woodrow IE Ball JT Berry JA 1987 A general expression for the control of the rate of photosynthetic CO_2_, fixation by stomata, the boundary layer and radiation exchange. In Progress in photosynthesis research (eds Biggins J), vol. 4, pp. 225–228. Dordrecht, The Netherlands: Kluwer Academic Publishers.

[37] Woodrow IE Ball JT Berry JA 1990 Control of photosynthetic carbon dioxide exchange by the boundary layer, stomata and ribulose 1,5-bisphosphate carboxylase/oxygenase. Plant Cell Environ. 13, 339–347. (10.1111/j.1365-3040.1990.tb02137.x)

[38] Baldocchi D 1994 An analytical solution for coupled leaf photosynthesis and stomatal conductance models. Tree Physiol. 14, 1069–1079. (10.1093/treephys/14.7-8-9.1069) 14967671

[39] Mansfield TA 1998 Oscar Victor Sayer Heath 26 July—16 June 1997. Biogr. Mems. Fell.R. Soc. 44, 219–235. (10.1098/rsbm.1998.0015)

[40] Sellers PJ *et al.* 1996 Comparison of radiative and physiological effects of doubled atmospheric CO_2_ on climate. Science 271, 1402–1406. (10.1126/science.271.5254.1402)

[41] Sellers PJ 1997 BOREAS in 1997: experiment overview, scientific results, and future directions. J. Geophys. Res. 102, 28 731–28 769. (10.1029/97JD03300)

[42] Cao L Bala G Caldeira K Nemani E Ban-Weiss G 2010 Importance of carbon dioxide physiological forcing to future climate change. Proc. Natl Acad. Sci. USA 107, 9513–9518. (10.1073/pnas.0913000107) 20445083 PMC2906877

[43] Betts RA Cox PM Lee SE Woodward FI 1997 Contrasting physiological and structural vegetation feedbacks in climate change simulations. Nature 387, 796–799. (10.1038/42924)

[44] Sellers P 1997 Modeling the exchanges of energy, water, and carbon between continents and the atmosphere. Science 275, 502–505. (10.1126/science.275.5299.502) 8999789

[45] Friedlingstein P 2006 Climate–carbon cycle feedback analysis: results from the C4MIP model intercomparison. J. Clim. 19, 3337–3353. (10.1175/JCLI3800.1)

[46] Sitch S 2008 Evaluation of the terrestrial carbon cycle, future plant geography and climate-carbon cycle feedbacks using five dynamic global vegetation models (DGVMs). Glob. Change Biol. 14, 2015–2039. (10.1111/j.1365-2486.2008.01626.x)

[47] Piao S 2013 Evaluation of terrestrial carbon cycle models for their response to climate variability and to CO_2_ trends. Glob. Change Biol. 19, 2117–2132. (10.1111/gcb.12187) 23504870

[48] Le Quéré C 2009 Trends in the sources and sinks of carbon dioxide. Nat. Geosci. 2, 831–836. (10.1038/ngeo689)

[49] Ciais P 2013 Carbon and other biogeochemical cycles. In Climate change 2013: the physical science basis. Contribution of Working Group I to the Fifth Assessment Report of the Intergovernmental Panel on Climate Change (eds Stocker TF Qin D Plattner G-K Tignor M Allen SK Boschung J Nauels A Xia Y Bex V Midgley PM). Cambridge, UK: Cambridge University Press.

[50] Buckley TN Mott KA 2013 Modelling stomatal conductance in response to environmental factors. Plant Cell Environ. 36, 1691–1699. (10.1111/pce.12140) 23730938

[51] Cowan I 2013 Fit, fitter, fittest: where does optimisation fit in? Silva Fenn. 36, 745–754.

[52] Medlyn BE Duursma RA Kauwe MG Prentice IC 2013 The optimal stomatal response to atmospheric CO_2_ concentration: alternative solutions, alternative interpretations. Agric. For. Meteorol. 182–183, 200–203. (10.1016/j.agrformet.2013.04.019)

[53] Sperry JS Hacke UG Oren R Comstock JP 2002 Water deficits and hydraulic limits to leaf water supply. Plant Cell Environ. 25, 251–263. (10.1046/j.0016-8025.2001.00799.x) 11841668

[54] Tardieu F Davies WJ 1993 Integration of hydraulic and chemical signalling in the control of stomatal conductance and water status of drought plants. Plant Cell Environ. 16, 341–349. (10.1111/j.1365-3040.1993.tb00880.x)

[55] Tuzet A Perrier A Leuning R 2003 A coupled model of stomatal conductance, photosynthesis and transpiration. Plant Cell Environ. 26, 1097–1116. (10.1046/j.1365-3040.2003.01035.x)

[56] Fisher RA Williams M Da Costa AL Malhi Y Da Costa RF Almeida S Meir P 2007 The response of an Eastern Amazonian rain forest to drought stress: results and modelling analyses from a throughfall exclusion experiment. Glob. Change Biol. 13, 2361–2378. (10.1111/j.1365-2486.2007.01417.x)

[57] Hickler T Smith B Sykes MT Davis MB Sugita S Walker K 2004 Using a generalized vegetation model to simulate vegetation dynamics in North eastern USA. Ecology 85, 519–530. (10.1890/02-0344)

[58] Ostle NJ 2009 Integrating plant-soil interactions into global carbon cycle models. J. Ecol. 97, 851–863. (10.1111/j.1365-2745.2009.01547.x)

[59] Boote KJ Jones JW White JW Asseng S Lizaso JI 2013 Putting mechanisms into crop production models. Plant Cell Environ. 36, 1658–1672. (10.1111/pce.12119) 23600481

[60] Long SP Ainsworth EA Leakey ADB Nosberger J Ort DR 2006 Food for thought: lower-than-expected crop yield stimulation with rising CO_2_ concentrations. Science 312, 1918–1921. (10.1126/science.1114722) 16809532

[61] Bernacchi CJ Kimball BA Quarles DR Long SP Ort DR 2007 Decreases in stomatal conductance of soybean under open-air elevation of [CO_2_] are closely coupled with decreases in ecosystem evapotranspiration. Plant Physiol. 143, 134–144. (10.1104/pp.106.089557) 17114275 PMC1761983

[62] Leakey ADB Ainsworth EA Bernacchi CJ Rogers A Long SP Ort DR 2009 Elevated CO_2_ effects on plant carbon, nitrogen, and water relations: six important lessons from FACE. J. Exp. Bot. 60, 2859–2878. (10.1093/jxb/erp096) 19401412

[63] MacAlister CA Ohashi-Ito K Bergmann DC 2007 Transcription factor control of asymmetric cell divisions that establish the stomatal lineage. Nature 445, 537–540. (10.1038/nature05491) 17183265

[64] Hu H 2010 Carbonic anhydrases are upstream regulators of CO_2_-controlled stomatal movements in guard cells. Nat. Cell Biol. 12, 87–93. (10.1038/ncb2009) 20010812 PMC2906259

[65] Engineer CB Ghassemian M Anderson JC Peck SC Hu H Schroeder JI 2014 Carbonic anhydrases, *EPF2* and a novel protease mediate CO_2_ control of stomatal development. Nature 513, 246–250. (10.1038/nature13452) 25043023 PMC4274335

[66] Dow GJ Bergmann DC Berry JA 2014 An integrated model of stomatal development and leaf physiology. New Phytol. 201, 1218–1226. (10.1111/nph.12608) 24251982

[67] Dow GJ Berry JA Bergmann DC 2014 The physiological importance of developmental mechanisms that enforce proper stomatal spacing in *Arabidopsis thaliana*. New Phytol. 201, 1205–1217. (10.1111/nph.12586) 24206523

[68] Dow GJ Bergman DC 2014 Patterning and processes: how stomatal development defines physiological potential. Curr. Opin. Plant Biol. 21, 67–74. (10.1016/j.pbi.2014.06.007) 25058395

[69] Keenan TF 2013 Increase in forest water-use efficiency as atmospheric carbon dioxide concentrations rise. Nature 499, 324–327. (10.1038/nature12291) 23842499

[70] Gedney N Cox PM Betts RA Boucher O Huntingford C Stott PA 2006 Detection of a direct carbon dioxide effect in continental river runoff records. Nature 439, 835–838. (10.1038/nature04504) 16482155

[71] Godfray HCJ 2010 Food security: the challenge of feeding 9 billion people. Science 327, 812–818. (10.1126/science.1185383) 20110467

[72] Ray DK Mueller ND West PC Foley JA 2013 Yield trends are insufficient to double global crop production by 2050. PLoS ONE 8, e66428 (10.1371/journal.pone.0066428) 23840465 PMC3686737

[73] Intergovernmental Panel on Climate Change (IPCC). 2013 Summary for policymakers. In Climate change 2013: the physical science basis. Contribution of Working Group I to the Fifth Assessment Report of the Intergovernmental Panel on Climate Change (eds Stocker TF Qin D Plattner G-K Tignor M Allen SK Boschung J Nauels A Xia Y Bex V Midgley PM). Cambridge, UK: Cambridge University Press.

[74] Berry JA Beerling DJ Franks PJ 2010 Stomata: key players in the Earth system, past and present. Curr. Opin. Plant Biol. 13, 232–239. (10.1016/j.pbi.2010.04.013) 20552724

